# Band-Pass Raman Spectroscopy Unlocks Compact Point-of-Care Noninvasive Continuous Glucose Monitoring

**DOI:** 10.1021/acs.analchem.5c01146

**Published:** 2025-12-04

**Authors:** Arianna Bresci, Youngkyu Kim, Miyeon Jue, Peter T. C. So, Jeon Woong Kang

**Affiliations:** † Laser Biomedical Research Center, G. R. Harrison Spectroscopy Laboratory, 2167 Massachusetts Institute of Technology, Cambridge, 02139 Massachusetts, United States; ‡ Apollon Inc., Gangseo-gu, Seoul 07795, Republic of Korea; § Department of Mechanical Engineering, 2167 Massachusetts Institute of Technology, Cambridge, 02139 Massachusetts, United States; ∥ Department of Biological Engineering, 2167 Massachusetts Institute of Technology, Cambridge, 02139 Massachusetts United States

## Abstract

Noninvasive blood glucose monitoring with precision comparable to standard invasive or minimally invasive methods has been a long-sought goal, especially as diabetes rates soar, with 592 million cases worldwide expected by 2035. Various optical and spectroscopic technologies have challenged noninvasive continuous glucose monitoring (CGM), but most methods fail to detect physiological levels or lack miniaturization for practical use. Based on our previous success in direct observation of glucose signals from *in vivo* skin, we developed a band-pass Raman spectroscopy method that enables noninvasive, physiological-level CGM in a compact device. Using off-axis 830 nm near-infrared illumination and intraspectrum reference, we eliminate most elastically scattered photons, revealing the glucose Raman signal through an amplified photodetector, while compensating for background variations. Our approach, validated on both tissue phantoms and *in vivo* human skin, overcomes bulky spectrometers and makes portable Raman-based CGM devices a reality.

## Graphical abstract

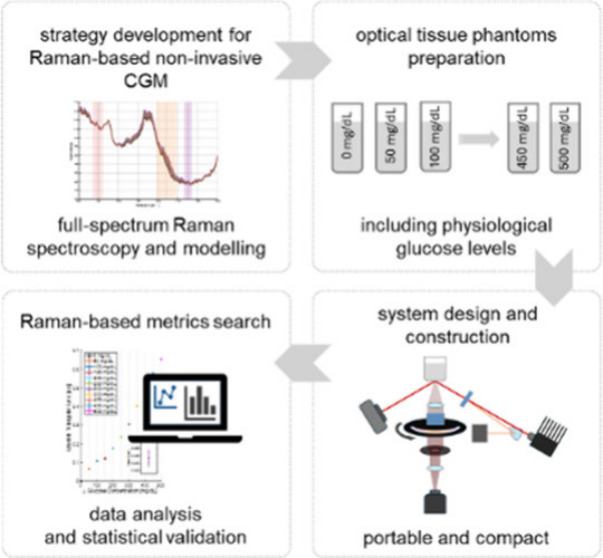

## Introduction

Diabetes has reached epidemic proportions worldwide, with projections indicating 592 million cases^1^ by 2035. Managing this chronic condition relies heavily on effective blood glucose monitoring, a cornerstone of diabetes treatment. Continuous glucose monitoring (CGM) systems have emerged as transformative tools not only for diabetes management but also for wellness tracking and athletic performance optimization.^1,2^ Despite their utility, traditional finger-pricking methods are impractical for continuous use, and current commercial CGMs, though effective, rely on minimally invasive microneedles to measure interstitial fluid (IF) glucose levels, falling short of true noninvasiveness, besides being costly due to sensor replacement needed every 10–14 days.^3^

Noninvasive approaches have sought to overcome these limitations through diverse technologies, which can be grouped into three subsets: direct, indirect and inference-based methods. Vibrational spectroscopy methods, including Raman,^4^ near-infrared (NIR),^5^ and mid-infrared^6^ spectroscopy, directly target the molecular signatures of glucose. Conversely, indirect techniques, such as photothermal and photoacoustic spectroscopy, leverage thermal or acoustic changes in tissue properties induced by glucose absorption.^7^ Similarly, optical coherence tomography^8^ and polarimetry^9^ measure refractive index (RI) changes and glucose chirality, respectively, while fluorescence-based methods use glucose-sensitive fluorophores.^10^ Emerging modalities like terahertz spectroscopy,^11^ microwave^12^ and radiofrequency^13^ sensing, ultrasound-assisted^14^ techniques, and bioimpedance spectroscopy^15^ exploit changes in tissue optical, electrical, or thermal properties. Other techniques, such as photoplethysmography^16^ and breath analysis,^17^ infer glucose levels through secondary measurements of physiological effects or glucose metabolism byproducts. These include alternative fluid sampling (tears, saliva, sweat) as a minimally invasive option.^18^ Many of these methods increasingly rely on artificial intelligence (AI) to process complex and noisy signals.^19^ However, dependence on black-box AI pipelines introduces challenges in explainability and generalizability, as they require extensive training and may not adapt well to diverse patient populations or conditions. Such limitations emphasize the importance of robust and high-specificity measurements based on physical models. Our band-pass Raman spectroscopy (BRS) approach addresses these challenges, offering a compact and noninvasive point-of-care solution for CGM.

## Experimental Section

### Strategy Development via Full-Spectrum Raman Spectroscopy and Tissue Phantom Modeling

We employed a home-built full-spectrum dispersive Raman spectroscopy system^20^ to record the Raman spectra of individual components of tissue phantoms: 20% glucose solution in water, 20% intralipid emulsion (IL), and phosphate-buffered saline solution (PBS). The analysis focused on the 600–1800 cm^–1^ region, known as the fingerprint region of the Raman spectrum, which captures the most prominent Raman peak of glucose at 1125 cm^–1^, as recently demonstrated by Kang et al.^21^ This is clear in simulations of the full Raman spectrum of no-glucose and high-glucose phantoms obtained via a linear combination of the spectra of individual components (Figure 1b) (Supplementary Note 1). We report 10 additional simulations of full spectra of tissue phantoms through physiological glucose concentrations ranging from 50 to 500 mg/dL in 50 mg/dL increments, from which the linear scaling of the Raman peak with glucose levels can be appreciated (Figure 1d and Supplementary Table 1). Instead of sampling redundant information through full-spectrum Raman spectroscopy, we identified three optimal spectral regions to be sampled via band-pass Raman spectroscopy. In addition to the glucose Raman peak at ∼1125 cm^–1^, we selected two sidebands at ∼950 cm^–1^ and ∼1175 cm^–1^ to compensate for background variations and serve as an intraspectrum reference. We opted for customized filters at 948.03/24.62 cm^–1^ (901.3/2 nm) and 1175.32/20.08 cm^–1^ (920.15/1.7 nm) for the sidebands, and 1120.12/50.11 cm^–1^ (915.5/4.2 nm) for the glucose peak (Supplementary Figure 1).

**Figure 1:**
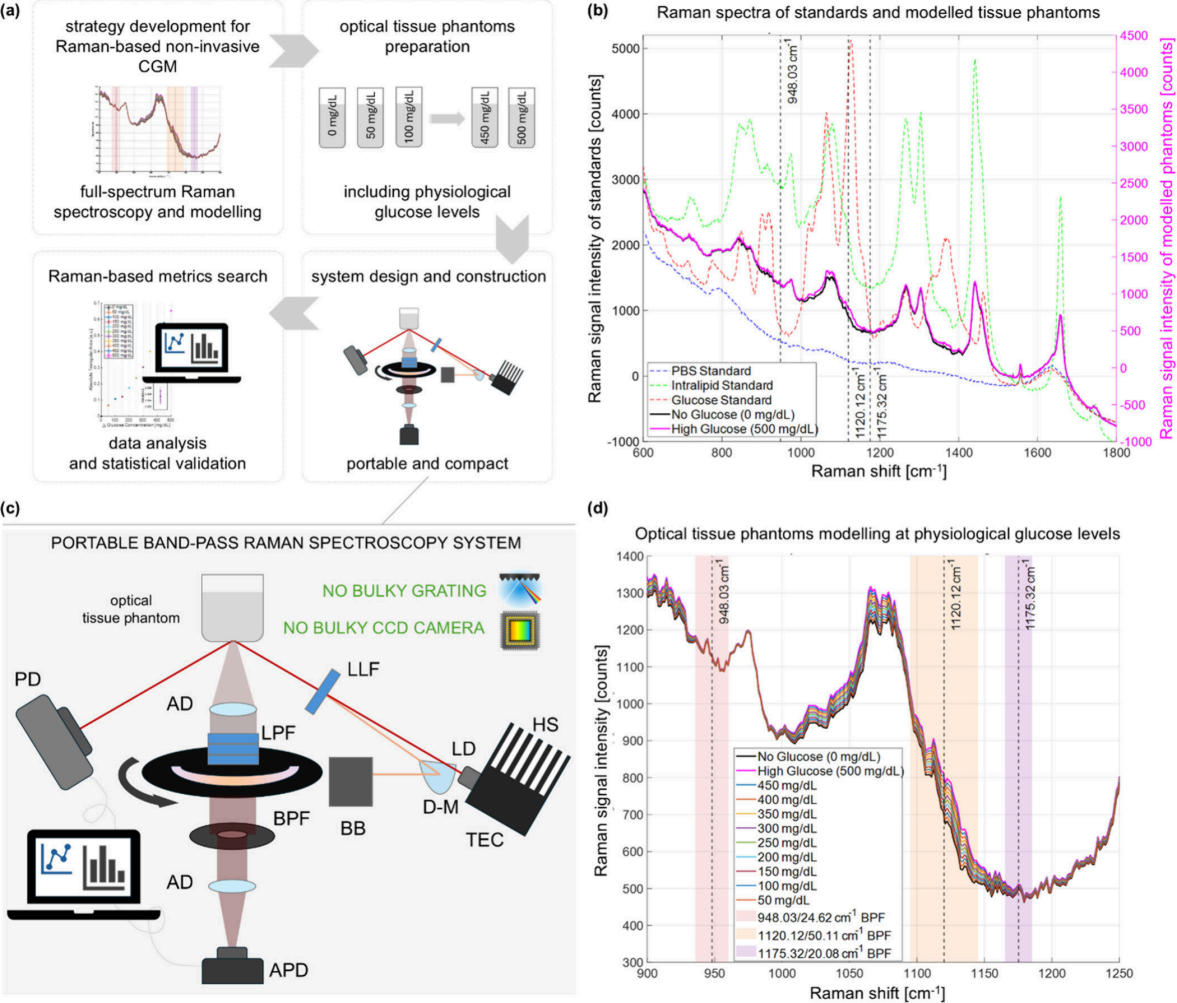
Pipeline for the development of compact BRS-based CGM. (a) Strategy development pipeline. (b) Measured full-spectrum Raman signal of individual components and modeled tissue phantom signals: PBS (blue line), 20% intralipid solution in water (green line); 20% glucose solution in water (red line); simulated no-glucose tissue phantom (black line); simulated high-glucose tissue phantom (pink line). (c) Scheme of the optical system for BRS. HS: heat sink; TEC: temperature control; D-M: D-shaped mirror; BB: beam blocker; LLF: laser line filter; AD: achromatic doublet; LPF: long-pass filter; BPF: band-pass filter; APD: amplified photodiode; PD: photodiode (d) Simulated Raman spectra around the Raman peak of glucose at 1125 cm^–1^ through 11 glucose levels. Shear areas indicate the chosen bands for BRS.

### BRS System Design and Construction

Our BRS system is designed to achieve compact, efficient, and precise glucose quantification through optimized optics (Figure 1c). It uses a collimated laser beam at 830.35 nm (Innovative Photonics Solutions, USA) with a laser diode current of 275 mA and a thermoelectric cooler (TEC) temperature set to 25 °C. A heat sink (HS) is mounted onto the laser diode to ensure thermal dissipation during operation, crucial when the optical setup is fully enclosed in a portable box. The laser beam illuminates the tissue phantom through a 170-μm-thick quartz window at an incidence angle of 60°, over an elliptical area approximately 0.5 mm × 1 mm in size. This configuration yields a delivered power of 91.5 mW, and an irradiance of 18.3 W·cm^–2^. The inclined illumination minimizes reflected photons leaking into the detection branch oriented normally to the sample surface. To spectrally purify the laser output, a laser line filter (Semrock, USA) is placed at a 1°–2° inclination thus preventing its back reflection from reentering the diode.

Scattered light from the sample is collimated through a 20 mm-focal-length achromatic doublet (Thorlabs, USA). Three long-pass filters (Semrock, USA) isolate red-shifted Raman photons from background signals, followed by a motorized filter wheel (Thorlabs, USA) housing the ultranarrow band-pass filters that rotate to allow for sequential BPR signal acquisition. Filtered Raman photons are then focused onto an amplified femtowatt silicon photoreceiver (Femto, Germany) using 20 mm-focal-length achromatic doublet lens (Thorlabs, USA). A photomultiplier amplified detector (Thorlabs, USA) collects reflected photons to be used as reference about laser power fluctuations or changes in the sample refractive properties. Both the APD and the PD are connected to a data acquisition (DAQ) board (National Instruments, USA) operating at 100 kHz, and a custom-designed MATLAB application is used for opto-mechanical control and signal acquisition. The system is mounted on breadboards and enclosed, resulting in a 31 × 27 × 21 cm^3^ compact portable configuration. A sample holder with a quartz window is mounted on top of the device providing convenient access for tissue phantom preparation.

### Signal Processing, Metric Search and Statistical Validation

Each glucose measurement takes only ∼36 s: 10 s of continuous data acquisition per band, preceded by a 3-s pause to minimize noise due to mechanical filter switch. DAQ samples are averaged every second (100,000 samples), producing raw data from the APD Raman signal (Figure 2a) and reference data from the PD signal (Figure 2b). To account for laser power fluctuations, raw data are divided by their reference, yielding corrected data (Figure 2c). Each band is weighted by its band-pass filter bandwidth, producing weighted data (Figure 2d). One can also target differential glucose concentration subtracting the lowest concentration signal (0 mg/dL) from the others, resulting in corrected adjusted data (Figure 2e) or corrected weighted adjusted data (Figure 2f).

**Figure 2:**
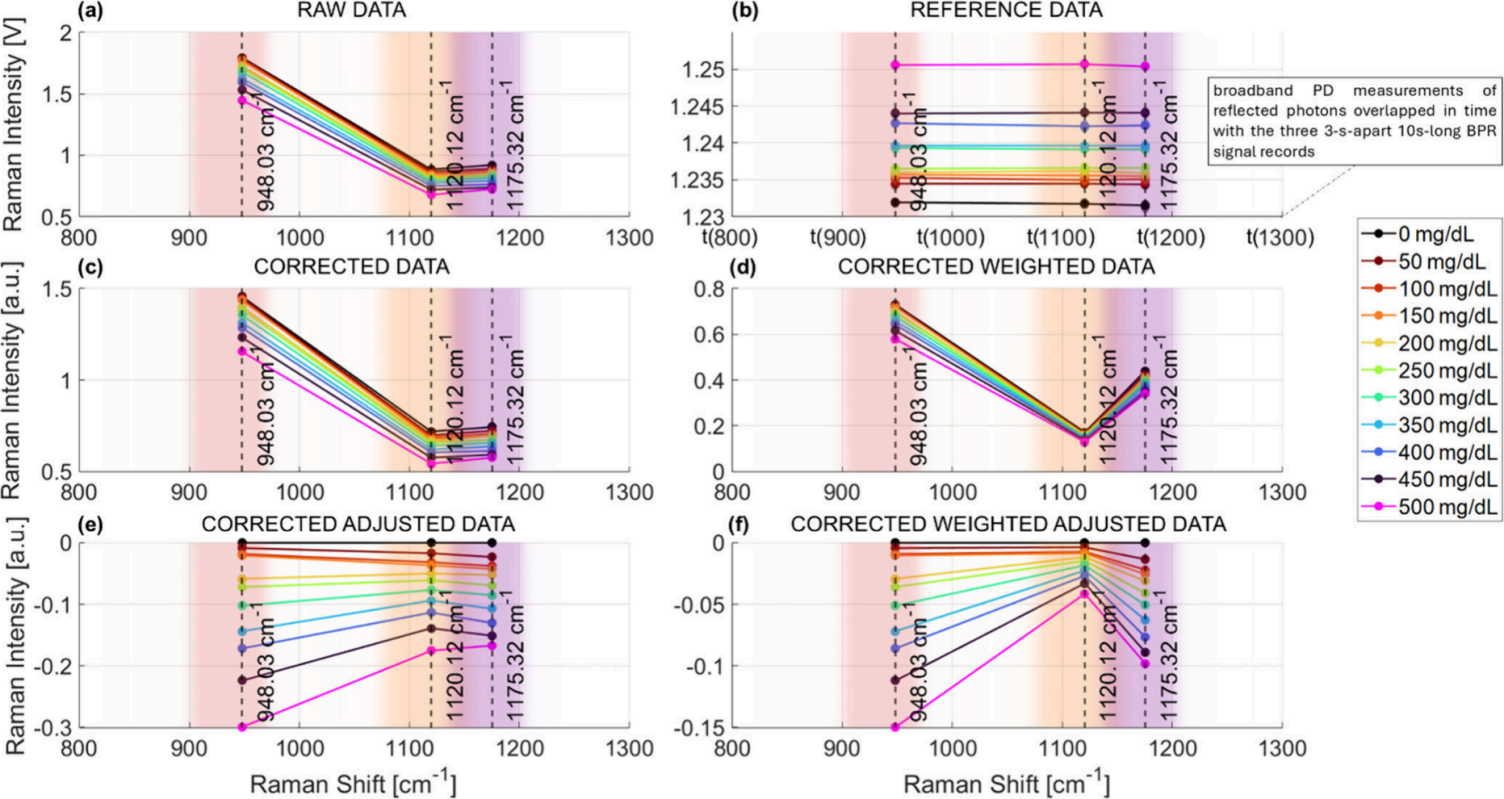
BRS glucose signal preprocessing steps. (a) Raw APD-detected Raman signals and (b) raw PD-detected reference signals, 1-s-averaged 10-s-long measurements. (c) Raw data are corrected by the reference signal. (d) Corrected data are weighted by the filter bandwidths. (e) Corrected data are adjusted by the lowest concentration signal. (f) Corrected weighted data are adjusted by the lowest concentration signal.

Raw data (Figure 2a) match the simulations (Figure 1d and Supplementary Figure 1): the 901.3 nm Raman band features a higher intensity compared to other bands, as higher background signal occurs (Figure 1d). In reference data (Figure 2b) we observe that higher glucose levels lead to a higher reflected intensity. As glucose concentration increases, the tissue phantom RI rises due to the higher optical density introduced by glucose molecules. This narrows the refractive index mismatch in the intralipid-PBS medium; more photons travel with minimal angular deviation and exit the phantom along near-specular trajectories. (Figure 2b). The photon flux available for Raman scattering diminishes, leading to a decrease in total Raman intensity through all the three bands (Figure 2a, Figure 3e). Despite this, the glucose signature remains quantifiable using sidebands as intraspectrum references.

**Figure 3:**
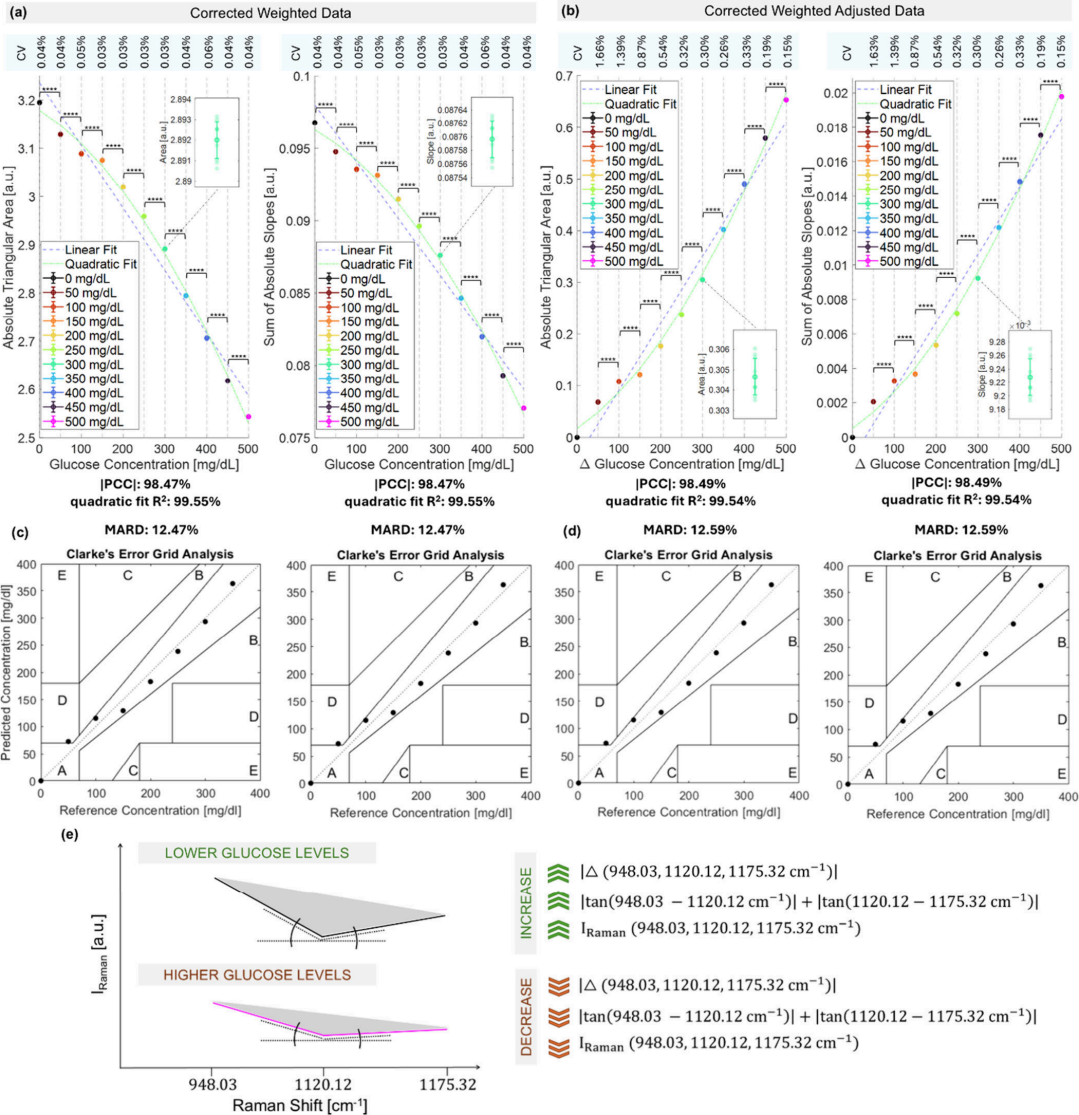
Metrics scaling proportionally with the glucose-specific Raman signal. Absolute area and sum of absolute slopes metrics calculated on Raman signals corrected weighted (a) and corrected weighted adjusted data (b). Statistically significant differences between glucose levels are evaluated via Student *t* testing, after assessing their normal distribution via the Lilliefors test (**** p-value <0.0001). Clarke error grid analysis of the quadratically calibrated Raman glucose, for corrected weighted data (c) and corrected weighted adjusted data (d). (e) Metrics formulas and their behavior at different glucose levels.

The metrics we compute are the absolute area encompassed by the signal at the three Raman bands and the sum of the two absolute slopes between the bands (Figure 3a,b) (Supplementary Note 2). When nonadjusted data are used (Figure 3a), the metrics scale with absolute glucose concentration, decreasing with higher glucose levels. This is because as glucose concentration increases the Raman peak of glucose rises in intensity and moves closer to the intensities of the higher sidebands. Conversely, for lower glucose concentrations, the metrics exhibit higher values, reflecting the greater contrast between the Raman glucose peak and the sidebands (Figure 3c). Clearly, this metrics behavior is typical when the measured BPR spectrum forms an inverse triangular shape, with the glucose peak as the lower intensity vertex (Figure 2a). Whether slightly positive or slightly negative triangular, the shape of the band-pass Raman spectrum is entirely acceptable and does not impact the reliability of the metrics (Supplementary Note 3). Metrics computed from differential glucose concentrations (Figure 3b) always exhibit direct proportionality with glucose levels, due to the differential nature of the processed data: higher glucose concentrations differ more from the lowest concentration used for adjustment, compared to lower concentrations (Figure 2f).

The Pearson correlation coefficient PCC between the metrics and glucose levels in corrected weighted data is 98.47%, while it is 98.49% in corrected weighted adjusted data (Figure 3a,b), indicating a strong linear relationship and suggesting consistency and reliability in tracking glucose. For both corrected weighted and corrected weighted adjusted data and for both metrics, the linear fit R^2^ is 97%, while the quadratic fit R^2^ is notably higher at 99.5% (Figure 3a,b). This indicates that the metrics follow a predominantly quadratic relationship with glucose levels in the voxel. The quadratic regression-based limit of detection^22–24^ is 37.53 ± 15.05 mg/dL (Supplementary Figure 3), comparable to or slightly below than the phantom glucose concentration spacing and below the physiological range. Building on this, we implement a quadratic calibration of the Raman-based metrics to evaluate glucose levels prediction. The results demonstrate that the mean absolute relative difference (MARD) between the predicted and actual glucose concentrations is 12.47% using corrected adjusted data (Figure 3c), and 12.59% using corrected weighted adjusted data (Figure 3d). A MARD below 15% is considered acceptable for CGM systems at a clinical level. Similarly, the Clarke error grid analysis, a method commonly applied for glucose levels up to 400 mg/dL, exhibits comparable results for both metrics and data preprocessing strategies, with most observations falling into the clinically acceptable A zone. Only one observation lies on the border within the D zone, associated with potentially dangerous clinical errors. This discrepancy may be attributed to sample preparation challenges: achieving homogeneity and precise concentrations is difficult at lower glucose levels through sequential dilutions.

### Clinical Intraskin Application to Humans

To evaluate the feasibility of our Raman-based CGM system in a clinical setting, we conducted a preliminary trial on a 27-years-old healthy (i.e., HbA1c = 5.04) male with a Fitzpatrick skin type II. All studies involving human subjects were approved by the Massachusetts Institute of Technology Committee On the Use of Humans as Experimental Subjects (COUHES# 23120011­74A001). The The participant was monitored over approximately 4 h and measurements were taken every 5 min using our Raman-based portable system on the nondominant forearm. Two needle-based CGMs – Abbott Freestyle Libre 3 and Dexcom G7 – were inserted in the dominant arm to record IF glucose levels every 5 min, while finger-pricking via a Nova Biomedical standard glucometer recorded blood glucose levels every 10 min. To induce dynamic changes in glucose levels, two 75 g glucose drinks (GlucoCrush, standard oral glucose tolerance test) were administered during the trial (Figure 4c). It should be noted that interstitial fluid glucose lags blood glucose by ∼5–15 min, a phenomenon accounted for in full-spectrum Raman-based^25,26^ and commercial CGMs. Here, we did not apply lag compensation to present unaltered optical readouts.

**Figure 4:**
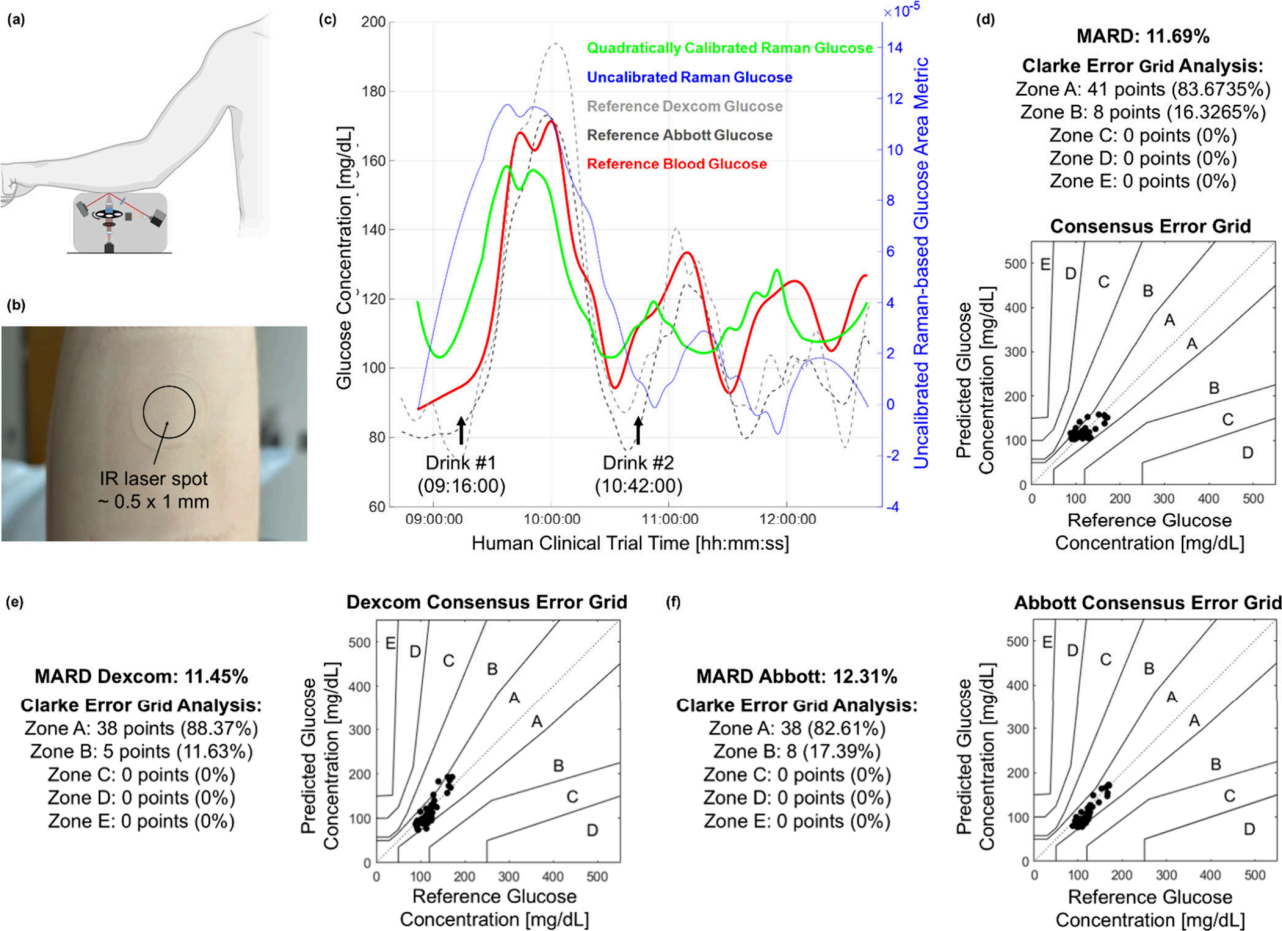
BRS-based portable system is used for *in vivo* intraskin CGM on humans. (a) Forearm and portable system configuration for clinical trials. (b) Picture of the spot of incidence of the 830.35 nm beam onto the participant skin after the trial, yielding a delivered power of 110 mW and an irradiance of 22.01 W·cm^–2^ (similar or lower than previous works in transdermal Raman spectroscopy^21,27,28^ ). No irritation was present, and no adverse event was reported in the following 10 months. (c) Glucose levels [mg/dL] in blood (red), in the IF via Abbott and Dexcom CGM devices (shades of gray), and by our calibrated BRS-CGM device (green). The uncalibrated area metric is referred to the left *y*-axis (blue). The timing of glucose drinks intake is reported. (d) Consensus error grid analysis of the BRS-CGM signal compared to the blood glucose levels. (e) Consensus error grid analysis of the Dexcom G7. (f) Consensus error grid analysis of the Abbott Freestyle Libre 3.

*In-vivo* clinical testing introduces significant challenges: participant movements over extended periods, variations in skin moisture, differences in the pressure of the forearm against the device, and melanin in the skin all contribute to signal variability. To address these complexities, we developed a preliminary data analysis strategy that includes baseline detrending to remove slowly varying background signals (Supplementary Figure 4). Glucose metrics were computed as described in the tissue phantom study from corrected weighted data, followed by a quadratic calibration onto blood glucose levels (Figure 4c and Supplementary Figure 2). The results of this preliminary trial are highly promising: the BRS-CGM system achieved a MARD of 11.69% (Figure 4d), the Dexcom G7 and the Abbott Freestyle Libre 3 achieved a MARD of 11.45% (Figure 4e) and 12.31% (Figure 4f). Our system shows comparable performance with needle-based commercial CGM devices. The Parkes (Consensus) error grid analysis further supports its reliability, with 100% of observations within the clinically accepted zones A and B, indicating clinically accurate results and benign deviations with no impact on therapeutic decisions (Figure 4d).

## Conclusion

Our portable and noninvasive BRS system enables targeted detection of the glucose Raman signature without the need to collect the full-spectrum signal. Avoiding bulky diffraction gratings and expensive CCD cameras typical of standard Raman spectroscopy methods, we introduce a significant improvement in size, complexity, and cost with respect to other noninvasive CGM devices.^3^ A systematic data processing pipeline focused on intraspectrum relative changes makes our solution outstandingly robust across varying experimental conditions. Preliminary clinical data from healthy humans establishes a strong foundation for future studies, even though expanding the sample size will be crucial to fully realize the potential of this point-of-care technology. Our streamlined system, featuring a total measurement time under 1 min, unlocks practical *in vitro* and *in vivo* CGM and holds the potential to transform glucose monitoring, offering portability, accessibility, accuracy and continuity for both clinical and personal health management applications.

## Supplementary Material



## Notes

Simulations of optical tissue phantoms full Raman spectra (Supplementary Note 1); Calculation of intraspectrum referenced glucose metrics (Supplementary Note 2); BPR spectral shape and metric scaling (Supplementary Note 3); Optical tissue phantoms modeling parameters (Supplementary Table 1); Transmission curves of the ultranarrowband BPFs impact on the recorded Raman signal (Supplementary Figure 1); Fully assembled device (Supplementary Figure 2); Regression-based quantification of the limit of detection (Supplementary Figure 3); Step-by-step preliminary data analysis pipeline for clinical trial data on heathy humans (Supplementary Figure 4). (PDF)
