# Supplementary material for: Band-Pass Raman Spectroscopy Unlocks Compact Point-of-Care Noninvasive Continuous Glucose Monitoring
Source: Anal Chem. 2025 Dec 4;97(49):27020–6. doi: 10.1021/acs.analchem.5c01146 (PMC12713608; doi:10.1021/acs.analchem.5c01146)

Supporting Information

Band-pass Raman spectroscopy unlocks compact point-of-care non-invasive continuous glucose monitoring

Arianna Bresci[1], Youngkyu Kim[2], Miyeon Jue[2], Peter T. C. So[1,3,4], Jeon Woong Kang[1]*

[1] Laser Biomedical Research Center, G. R. Harrison Spectroscopy Laboratory, Massachusetts Institute of Technology, Cambridge, 02139 MA, USA

[2] Apollon Inc., Gangseo-gu, Seoul 07795, Republic of Korea

[3] Department of Mechanical Engineering, Massachusetts Institute of Technology, Cambridge, 02139 MA, USA

[4] Department of Biological Engineering, Massachusetts Institute of Technology, Cambridge, 02139 MA, USA

Supporting Information contents:

- Simulations of optical tissue phantoms full Raman spectra (Supplementary Note 1)
- Calculation of intra-spectrum referenced glucose metrics (Supplementary Note 2)
- BRS spectral shape and metric scaling (Supplementary Note 3)
- Optical tissue phantoms modelling parameters (Supplementary Table 1)
- Transmission curves of the ultra-narrowband BPFs impact on the recorded Raman signal (Supplementary Figure 1)
- Fully assembled device (Supplementary Figure 2).
- Regression-based quantification of the limit of detection (Supplementary Figure 3).
- Step-by-step preliminary data analysis pipeline for clinical trial data on heathy humans (Supplementary Figure 4)

Supplementary Note 1. Simulations of optical tissue phantoms full Raman spectra.

To simulate realistic tissue phantom full Raman spectra, we applied a linear combination of the Raman spectra of the three components (Fig. 1b):

$$I_{simulated}(\nu) = \alpha \cdot I_{glucose}(\nu) + \beta \cdot I_{intralipid}(\nu) + \gamma \cdot I_{PBS}(\nu)$$

where α, β, and γ represent the respective weight fractions of glucose (Sigma-Aldrich, USA, product code: 49163), IL (Sigma-Aldrich, USA, product code: I141), and PBS (Gibco, USA, product code: 10010023). The weights of the three components were adjusted accordingly in the simulated tissue phantoms to reflect different glucose concentrations and produce their expected Raman spectra. Using this model, we simulated the Raman spectrum of a no-glucose tissue phantom (25% intralipid emulsion and 75% PBS) and a high-glucose tissue phantom with 500 mg/dL glucose (maintaining a constant 25% intralipid emulsion). Analysis of the simulated spectra confirmed that the most prominent glucose Raman peak at 1125 cm^{-1} effectively distinguishes no-glucose and high-glucose conditions, with a clear spectral separation (Fig. 1b).

Supplementary Note 2. Calculation of intra-spectrum referenced glucose metrics.
The metrics computed from the processed data include the absolute area encompassed by the signal at the three Raman bands, namely:

$$Area\ metric = \frac{1}{2}\left|\left(948.03\left(I_{1120.12} - I_{1175.32}\right) + 1120.12\left(I_{1175.32} - I_{948.03}\right) + 1175.32\left(I_{948.03} - I_{1120.12}\right)\right)\right|,$$

and the sum of the two absolute slopes between the bands, namely:

$$Slope\ metric = \left|{}^{I_{1120.12} - I_{948.03}}\!/_{1120.12 - 948.03}\right| + \left|{}^{I_{1175.32} - I_{1120.12}}\!/_{1175.32 - 1120.12}\right|$$

as shown in Fig. 3a,b. To mitigate noise, the 10-second averaged signals from the sidebands and the 1-second averaged signal from the Raman glucose band are used for every 10-second band measurement. Metrics are computed after weighing data for the different filter bandwidths at the three Raman bands. This correction effectively reduces the standard deviation of the metric values, successfully unraveling their proportionality with respect to the glucose levels. This results in a coefficient of variation (CV) < 0.1% and CV < 1.5% for metrics derived from corrected weighted data and corrected weighted adjusted data, respectively (the CV statistically measures the relative dispersion of data points around the mean). Naturally, CV values increase in adjusted data, in which the variation is affected by both the noise of the current measurement and the noise of the lowest glucose concentration measurement used for the adjustment.

Supplementary Note 3. BRS spectral shape and metric scaling.

The shape of the band-pass Raman spectrum, whether slightly positive or slightly negative triangular, is entirely acceptable and does not impact the reliability of the metrics. This variation arises from a combination of instrumental factors, including the bandwidth and transmission efficiency of the band-pass filters and the quantum efficiency of the detector at different photon energies, as well as the scattering properties dictated by the tissue phantom formula. However, the metrics remain robust to these variations, as their proportional relationship to glucose levels is preserved. If such tissue phantom or system changes result in a positive triangle-shaped BRS spectrum, the same metrics can be applied but will scale directly with absolute glucose levels instead.

Supplementary Table 1. Optical tissue phantoms modelling parameters. To simulate realistic tissue phantom spectra, we used a linear combination of the Raman spectra of the three standard components, namely, a 20% glucose solution in water, a 20% IL emulsion, and PBS: $I_{simulated}(\nu) = \alpha \cdot I_{glucose}(\nu) + \beta \cdot I_{intralipid}(\nu) + \gamma \cdot I_{PBS}(\nu)$. In this equation, α, β, and γ represent the respective weight fractions of glucose, IL emulsion, and PBS. We adjusted the weights of the three components accordingly in the simulated tissue phantoms to reflect different glucose concentrations and produce their expected Raman spectra. Using this model, we simulated the Raman spectrum of a no-glucose tissue phantom (25% intralipid emulsion and 75% PBS) and a high-glucose tissue phantom with 500 mg/dL glucose (maintaining a constant 25% intralipid emulsion), as well as 10 additional glucose concentrations ranging from 50 mg/dL to 500 mg/dL in 50 mg/dL increments.

Glucose Concentration (mg/dL)	α (Glucose)	β (Intralipid)	γ (PBS)
0 (no glucose)	0	0.25	0.75
500 (high glucose)	0.2	0.25	0.55
450	0.18	0.25	0.57
400	0.17777	0.25	0.57223
350	0.175	0.25	0.575
300	0.171433	0.25	0.578567
250	0.166667	0.25	0.583333
200	0.16	0.25	0.59
150	0.15	0.25	0.6
100	0.133333	0.25	0.616667
50	0.1	0.25	0.65

Supplementary Figure 1. Transmission curves of the ultra-narrowband BPFs impact on the recorded Raman signal. (a) Ultra-narrowband BPFs transmission curves measured and averaged over a 10 pieces batch (lines) versus datasheet specifics (shear areas). The measured transmission curves appear as larger in bandwidth and show a slight shift in terms of central bandwidth. More in detail, the 948.03 cm^{-1} BPF features a measured lower Raman Shift (at 50% max transmission) at 901.80 cm^{-1} and an upper Raman Shift (at 50% max transmission) at 970.14 cm^{-1}, resulting in a 936/68.35 cm^{-1} BPF; the 1120.12 cm^{-1} BPF features a measured lower Raman Shift (at 50% max transmission) at 1076.52 cm^{-1} and an upper Raman Shift (at 50% max transmission) at 1152.00 cm^{-1}, resulting in a 1114.26/75.49 cm^{-1} BPF; the 1175.32 cm^{-1} BPF features a measured lower Raman Shift (at 50% max transmission) at 1137.75 cm-1 and an upper Raman Shift (at 50% max transmission) at 1202.30 cm^{-1}, resulting in a 1170.03/64.55 cm^{-1} BPF. **(b)** Considering the datasheet BPFs transmission curves at the selected wavelengths (while still excluding any detector quantum efficiency effect and other instrument parameters), the shape of the BRS spectrum recorded appears as upright triangular. The recorded Raman signal intensity is simulated as an integration of the area below the curve of the simulated tissue phantoms full Raman spectrum in Fig. 1d. **(c)** When considering the measured BPFs transmission curves, the shape of the BRS spectrum more closely resembles the inverted triangular shape of the experimental one in tissue phantoms, with the central peak significantly decreasing in relative intensity compared to the sidebands intensity.

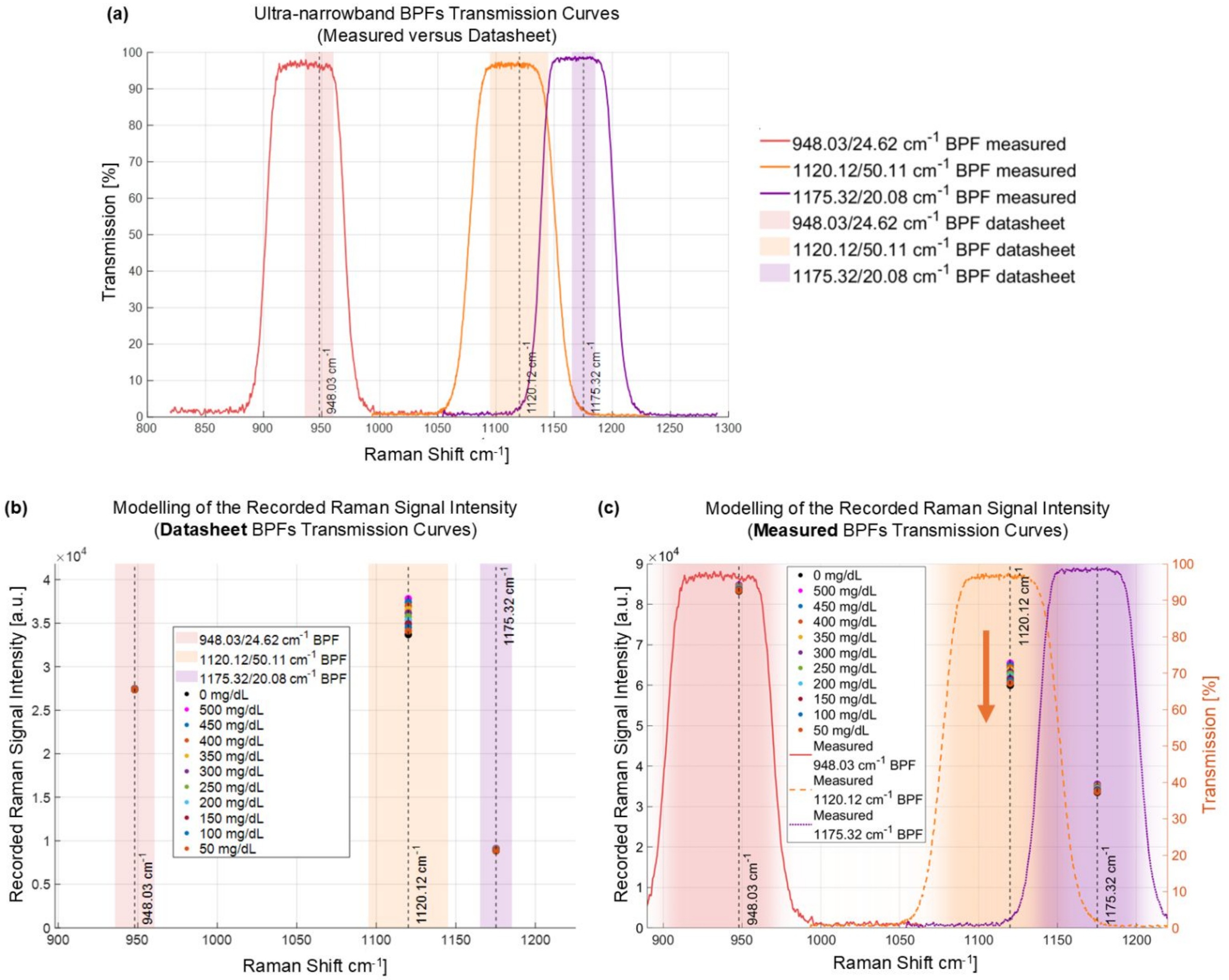

Supplementary Figure 2. Fully assembled device. Pictures of the compact, portable BRS-CGM device, including the aluminum case for shielding stray light and an arm holder interface for clinical trials on human subjects. The device dimensions are 31 × 27 × 21 cm. The dev ice weight, including the case but excluding power suppliers, is ~ 10 kg.

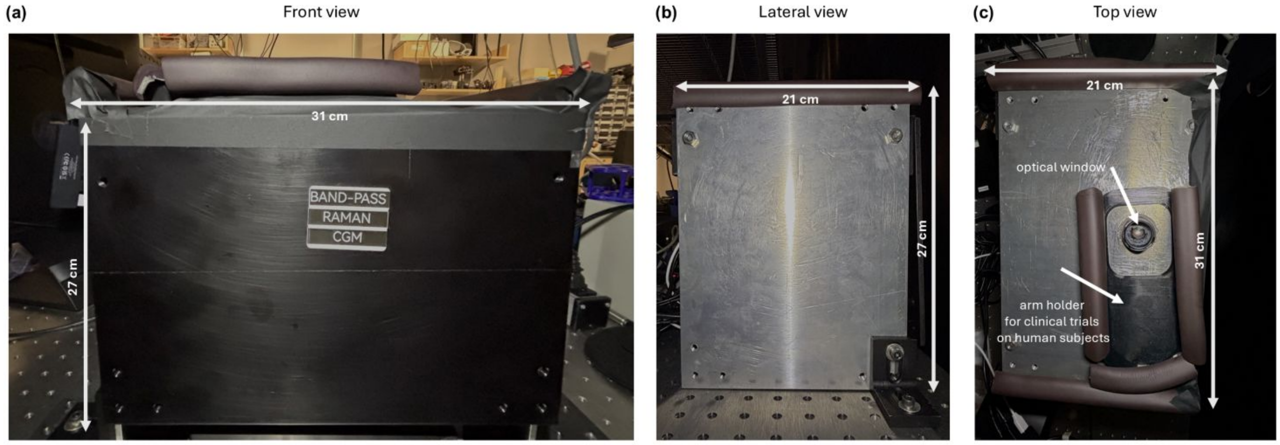

Supplementary Figure 3. Regression-based quantification of the Limit of detection (LoD). We quantified sensitivity using the regression-based framework commonly applied in analytical chemistry, where the instrument response is modeled as linear, $y = a + bx$, and $LoD = 3\sigma_{res}/|b|$, with σ_{res} the residual standard deviation, and b being the slope or sensitivity[22]. This basic formulation is widely accepted but assumes a linear calibration slope. In our case, BRS metrics exhibit a quadratic dependence on glucose concentration (*i.e.*, quadratic fit R^2 = 99.55% for area and slope metrics), and a purely linear LoD calculation therefore results in an overly conservative estimate. To address this, we extended the LoD framework to a quadratic fit, $y = a + bx + cx^2$. Instead of a single sensitivity value b, now we have a local sensitivity given by the derivative $b + 2cx$, and $LoD(x) = 3\sigma_{res}/|b+2cx|$[22–24]. We summarized the result across our experimental range as LoD = 37.53 mg/dL ± 15.05 mg/dL. Despite the conservative use of $3\sigma_{res}$ rather than $2\sigma_{res}$, this LoD estimate further confirms that the actual detection capability of our BRS system is comparable to or slightly below the phantom spacing used in the dilution series and falling well below the physiological range (80 – 160 mg/dL). **(a)** Quadratic fit of the absolute area metric using corrected weighted data; **(b)** quadratic fit of the absolute sum of slopes metric using corrected weighted data; **(c)** The mean LoD for the absolute area metric from weighted adjusted data, in the 0 – 500 mg/dL glucose range, is 37.53 mg/dL using $3\sigma_{res}$ and 25.02 mg/dL using $2\sigma_{res}$; **(d)** The mean LoD for the absolute sum of slopes metric from weighted adjusted data in the 0 – 500 mg/dL glucose range, is 37.53 mg/dL using $3\sigma_{res}$ and 25.02 mg/dL using $2\sigma_{res}$.

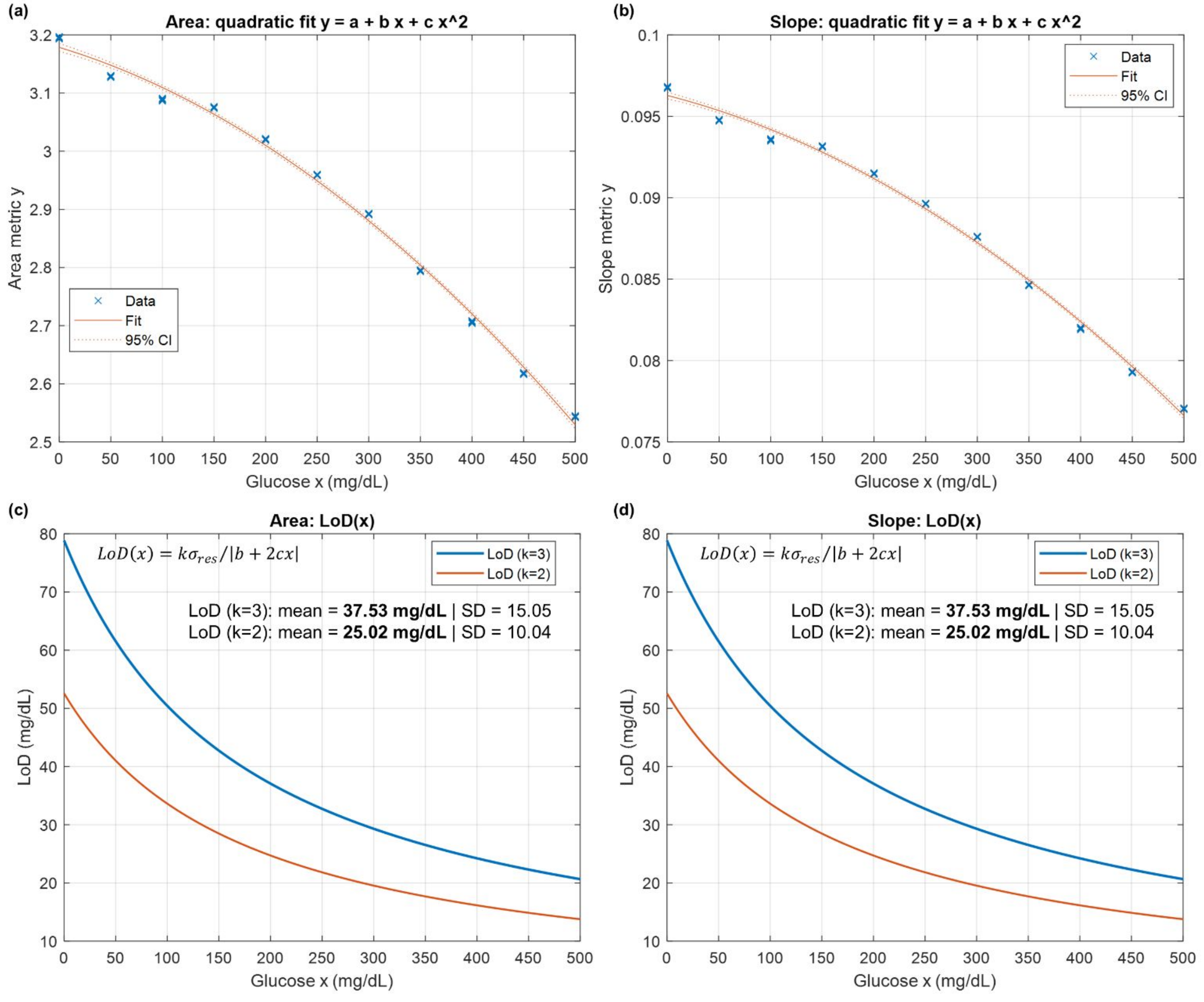

Supplementary Figure 4. Step-by-step preliminary data analysis pipeline for clinical trial data of heathy humans. (a) Reference glucose levels are measured both in the IF, via two needle-based commercially available sensors (*i.e.*, Abbott Freestyle 3 and Dexcom G7 CGMs) in the upper dominant arm of the participant, sampling every 5 minutes, and directly in blood via fingerpicking through a Nova Biomedical standard glucometer, sampling every 10 minutes. The curves shown are spline interpolations. **(b)** Similarly, the BRS portable system is used to quantify glucose levels in the IF, with a sampling period of 5 minutes. Analogously to the data processing pipeline developed for tissue phantoms, two metrics are calculated: the absolute area under the Raman peak of glucose (first column), and the sum of absolute slopes of the Raman peak of glucose (second column). Additionally, due to the complexity of the scattering background given by living human skin, the slope of the sidebands (excluding the signal at the glucose peak) is calculated to account for background variations (third column). To mitigate noise and smooth the interpolated Raman-based metric curves over time, a second-order Savitzky-Golay filter with a window size of 67 points is applied. **(c)** Most likely due to the photobleaching of skin pigments, we observe a smoothly varying background curve, on top of which the metric oscillations sit. We remove such background through baseline detrending via the adaptive smoothness partial least squares algorithm employing the following parameters: smoothness parameter = 10^5; order = 2; decay coefficient = 2; maximum number of iterations = 200; convergence threshold = 10^{-4}. **(d)** To compensate for the background variations and limit the impact of background-only dynamics to the Raman peak-related metrics of area and slope, we conclude the analysis by subtracting the background metric (third column) from the absolute area metric (first column) and from the sum of absolute slopes metric (second column).

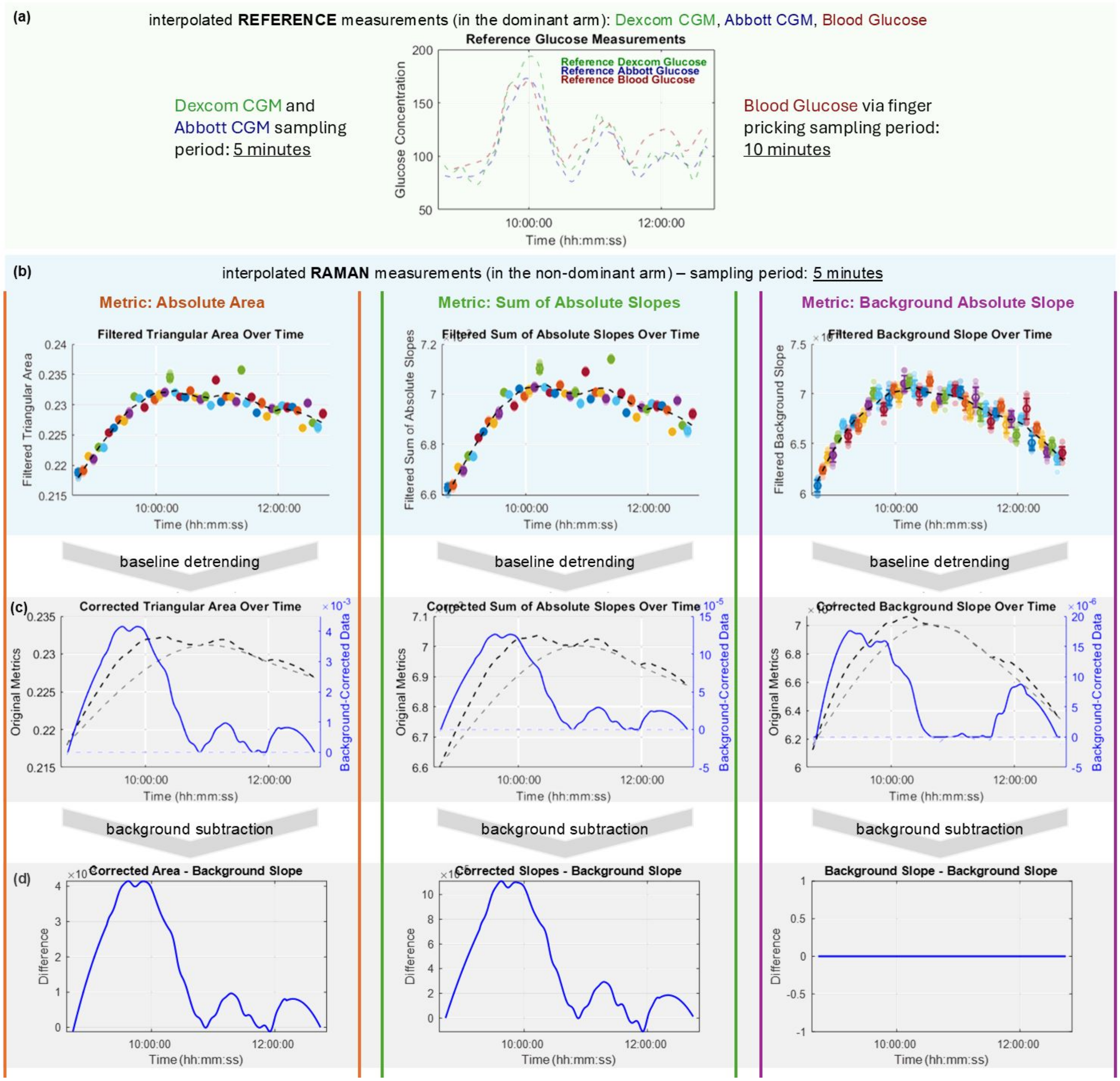

Supplement: Supplementary file 1 [file ac5c01146_si_001.pdf]
